# The systemic immune-inflammatory index in high-risk patients with hypertension: a cross sectional-study

**DOI:** 10.1590/1516-3180.2024.0301.R1.14072025

**Published:** 2025-09-19

**Authors:** Francelise Susan Mihara Bettanin, Marcelo Rodrigues Bacci

**Affiliations:** I Student, Programa de Pós-Graduação em Ciências da Saúde, Centro Universitário Faculdade de Medicina ABC; Nurse, Centro Universitário Faculdade de Medicina ABC, Santo André (SP), Brazil.; II Professor, Centro Universitário Faculdade de Medicina ABC, Santo André (SP), Brazil.

**Keywords:** Biomarkers., Risk factors., Kidney diseases., Hypertension., Cardiovascular diseases., Arterial hypertension., Inflammation., Systemic immune-inflammatory index.

## Abstract

**INTRODUCTION:**

Essential hypertension is an important health condition responsible for conditions such as heart attack, stroke, and kidney disease. Traditional risk factors and their control are important for reducing mortality. Inflammation caused by organ damage plays a role in the undesirable outcomes of hypertension. Biomarkers, such as the systemic immune-inflammatory index (SII), are predictors, but their relationship with risk factors is poorly understood.

**OBJECTIVE:**

To evaluate the correlation between the SII and risk factors in patients with hypertension.

**METHODS:**

This cross-sectional study was conducted in 2020 in Bahia with hypertensive patients from an outpatient clinic. We collected demographic and clinical data such as age, body mass index, and the following biomarkers: low-density lipoprotein level, glomerular filtration rate, interleukin 6 level, C-reactive protein level, neutrophil/lymphocyte ratio (NLR), hemogram, creatinine level, urea level, ferritin level, and vitamin D level.

**RESULTS:**

A total of 61 patients, most of them black women, participated in the study. The prevalence of type 2 diabetes was 19%, and there was no prevalence of stroke or heart attacks. According to the Framingham risk index, a large part of the sample presented high and very high risks. The bivariate analysis between SII and NLR was positive. Multivariate analysis showed that age, renal function, and NLR were positively correlated with the SII. The patients’ Framingham risk did not correlate with the SII.

**CONCLUSIONS:**

Inflammation is important for vascular damage in arterial hypertension caused by increased oxidative stress. We evaluated SII and NLR as indices of correlation with risk factors. The SII is a lowcost tool that can be used to screen for chronic conditions, such as hypertension. In summary, higher SII was positively associated with older age and worse renal function in patients with high-risk hypertension.

## INTRODUCTION

 Hypertension is a chronic non-communicable disease characterized by permanently elevated blood pressure. It is a complex syndrome influenced by genetic, epigenetic, environmental, and social variables, and is linked to cardiovascular disease (CVD).^1,2^ In the Americas, the condition affects over 25% of women and 40% of men.^1^ In Brazil, a national pool inquiry reported a prevalence of 23% of hypertension in young adults in 2013, reaching 47% in adults older than 74 years. Despite a reduction in the age-adjusted mortality model in the last three decades due to public prevention programs, the most vulnerable and economically disadvantaged population had a lesser impact on these policies in the northern and northeastern regions of the country.^2^ Data from the Brazilian Cardiovascular Statistics Registry show that CVD has been the most important cause of death in Brazil in the last 30 years, with coronary disease as the leading cause of CVD.^3^

 Higher blood pressure is linked to the development of atherosclerosis, a contributing factor to CVD.^3^ Adequate control of risk factors such as obesity, dyslipidemia, smoking, glycemic control, and sedentarism can prevent around 75% of CVD.^2^ The underlying physiological mechanism between CVD and hypertension is characterized by persistent inflammation and microcirculatory abnormalities.^ 3,4^ Biomarkers associated with chronic inflammation play a direct role in the development of atherosclerosis. These biomarkers include C-reactive protein (CRP), interleukins, and primary hematological indices such as the neutrophil-lymphocyte ratio (NLR), the platelet-lymphocyte ratio (PLR), and the systemic immune-inflammatory index (SII) which measures the balance between the inflammatory, immunological, and thrombotic statuses.^4-6^ The SII is calculated using the formula: (platelets × neutrophils) ÷ total lymphocyte levels.^7^ The higher the index, the worse the inflammation status. The SII outperforms indices such as the NLR in predicting some types of cancer.^8^ Additionally, the SII can predict unfavorable cardiac outcomes and the standard uncontrolled risk factors with a reduced cost following coronary intervention.^4,5^

 The study aimed to evaluate the impact of the traditional CVD risk factors among adults with hypertension and its correlation with the SII in a hypertension public facility unit care. 

## METHODS

### Study design

 This study employed a cross-sectional design utilizing quantitative and population-based methods. This study was conducted in the Barreiras district of northeastern Brazil. This district is responsible for a local gross domestic product (GDP) of approximately US$ 1,400,000, compared to the overall Brazilian GDP of approximately US$ 2,126 trillion.^9^

### Study location and period

 All participants in the hypertension follow-up program were invited to participate in the study, which spanned from April 2019 to January 2020, before the beginning of the COVID-19 pandemic in Barreiras, Bahia. The health unit in Barreiras is a countryside reference in Bahia that diagnoses, treats, and monitors patients with hypertension. 

### Study population and eligibility criteria

 Participants who were both over the age of 18 and diagnosed with hypertension were included in the study after providing written consent. Individuals who had received cancer therapy within the past five years, individuals with hepatitis and HIV, individuals with rheumatologic illnesses, pregnant women, and individuals who regularly used steroids were excluded from the study. 

### Data collection

 The data were collected between April 2019 and January 2020. The demographic data of each patient and risk factors for CVD were collected. The risk factors studied included dyslipidemia, diabetes, smoking, obesity, and CVD onset. A standard blood pressure measurement was used to define the arterial hypertension stage according to the Brazilian arterial hypertension guidelines, aligned with the American Heart Association recommendation.^2^

 The estimated glomerular filtration rate (eGFR) was evaluated with the serum creatinine result and the CKD-EPI equation.^10^ Chronic kidney disease (CKD) diagnosis followed the Kidney Disease Global Initiative Outcomes (KDIGO) guideline with a persistent eGFR below 60 ml/min/1,73 m^2^ for more than twelve weeks.^11^ The Framingham stratification CVD risk score and the inflammatory condition assessed the cardiovascular risk with the hs-CRP, interleukin-6 (IL-6), the NLR, and the SII.^6, 12^

### Data analysis and statistical analysis

 The data analysis began in February 2020. Data distribution was checked using the Kolmogorov-Smirnov test. The variables of height, waist circumference, LDL, IL6, hs-CRP, and creatinine presented a non-homogeneous distribution, described as the median, 25th percentile, and 75th percentile. Non-parametric tests were used to analyze variables with different distributions. Bivariate relationships between quantitative variables were analyzed using Spearman’s correlation test. A significance level of 0,05 (5%) was used in this study. 

 We performed multivariate analysis using multiple linear regression to evaluate the influence of age, body mass index, LDL, eGFR, IL6, hs-CRP, and NLR on the SII. Bootstrapping procedures (1000 resamples; 95% CI BCa) were carried out to obtain better reliability of the results, correct deviations from the normality of the sample distribution, and to present a 95% confidence interval (95% CI) for the Multiple Linear Regression.^13^ The selection of the Enter method made it possible to run the regression test in conjunction with the bootstrapping procedure. 

 An exploratory analysis was performed to correlate the Framingham risk score, stratified into low-, intermediate-, high-, and very high-risk groups, with the SII. Framingham risk strata were considered ordinal variables. We divided the four risk strata into two groups: low + intermediate risk and high + very high risk, and used Mann–Whitney analysis to evaluate the correlation with the SII. 

### Ethical and legal aspects of the research

 This study followed the STROBE systematization for cross-sectional studies (REF) and received local ethics approval (no. 3.286.842). Data were entered using Microsoft Excel software and the statistical package was SPSS v. 21.0 (IBM, Armonk, New York). 

## RESULTS

 The demographic and laboratory parameters of the patients are shown in **Table 1**. The 61 participants had a median age of 58 years, with a predominance of women (56%) of black ethnicity and without chronic kidney disease. No patient had a history of stroke, acute myocardial infarction, or secondary hypertension. The prevalence of type 2 diabetes was 19,6%, and 46% had dyslipidemia. The mean fasting glucose level of the sample was 111,6 mg/dl, indicating reasonable glycemic control. Only 13% reported smoking tobacco cigarettes. The hypertensive sample showed that 71% patients received angiotensin enzyme converting inhibitors (ACEi) or angiotensin receptor blockers (ARB) as renin-angiotensin-aldosterone system inhibition (RAASi). 

**Table 1:** Baseline characteristics, anesthetic management, and postoperative residual sedation in the PACU

**Variables**		**Participants (n = 61)**		
		**Median**	**Percentile 25**	**Percentile 75**
Age (years)		58	51	65
Waist (cm)		95	89	102
BMI (kg/m^2^)		27,1	25,2	30,8
Sex				
	*Male*		27 (44,3%)	
	*Female*		34 (55,7%)	
Ethnicity				
	*White*		6 (9,8%)	
	*Not white*		55 (90,2%)	
Alcohol consumption				
	*Yes*		12 (19,7%)	
	*No*		49 (80,3%)	
Hypertensive drugs				
	*None*		2 (3,3%)	
	*1 medicine*		19 (31,1%)	
	*2 medicines*		20 (32,8%)	
	*3 medicines*		11 (18%)	
	*4 or more medicines*		9 (14,8%)	
Hemoglobin (g/dl)		14	12,7	15,25
Hematocrit (%)		41,9	39,4	45,3
Creatinine (mg/dl)		0,89	0,73	1,08
Urea (mg/dl)		29	23	34
NLR		1,6	1,18	2,07
IL6 (pg/ml)		2,24	1,53	3,56
Ferritin (ng/ml)		154	99,65	207
Vitamin D (ng/ml)		25,5	21,75	29,7
hs-CRP (mg/l)		0,15	0,06	0,45
LDL (mg/dl)		107,5	90	140
eGFR (ml/min/1,73 m^2^ )		91,2	77,3	111,8
SII		371,2	249,04	504,56

BMI: body mass index; IL6: interleukin 6; hs-CRP: high sensitivity C-reactive protein; LDL: low-density lipoproteins; eGFR: estimated glomerular filtration rate; SII: systemic immune-inflammatory index.

 The results of the inflammatory tests, such as hs-CRP, SII, NLR, and IL6, showed that the cohort of patients presented a median hs-CRP result of 0,21 and a median SII result of 371,20. The Framingham CVD risk score was calculated among the sample individuals, revealing nine patients with low risk, 13 with intermediate risk, 37 with high risk, and two with very high risk. There was no significant difference between the risk stratification evaluation and the patient’s sex. 

**Table 2** shows Spearman’s correlation test between the dependent variable, SII, and the rest of the parameters. There was no correlation between SII and the other laboratory variables, only with the NLR. 

**Table 2:** Relationship between inflammatory markers and the systemic immune- inflammatory index

	**Correlacion coefficient (p)**
Age (years)	−0,076 (0,561)
BMI (kg/m^2^)	0,01 (0,938)
LDL (mg/dl)	0,031 (0,813)
eGFR (mL/min/1,73 m^2^)	−0,19 (0,143)
IL6 (pg/ml)	0,19 (0,166)
hs-CRP (mg/l)	−0,021 (0,873)
NLR	0,719* (< 0,001)

* Spearman correlation coefficient with P < 0.01

BMI: Body mass index; LDL: lowdensity lipoproteins; eGFR: estimated glomerular filtration rate; IL6: interleukin 6; hs-CRP: high-sensitivity C-reactive protein.

 The multivariate linear regression results demonstrated that the independent variables had a substantial impact on the SII (F (7, 46) = 110,863, P < 0.0001; R2 adjusted = 0,447). The coefficients for the three predictors that affected the SII, age, eGFR, and NLR are shown in **Table 3**. The dependent variable was not affected by the other evaluated variables. The predictive equation for this model was: SII = 539,88 −4,32 × Age −3,59 × eGFR + 193,78 × NLR. 

**Table 3:** Multivariate analysis of factors that influence the dependent variable Systemic inflammatory index

	**B**	**Beta coefficient**	**t**	**p**	**95% CI for B**	
					**Inferior limit**	**Upper limit**
(Constant)	539,88		2,07	0,04	14,52	1065,23
Age	−4,32	−0,26	−2,07	0,04	−8,53	−0,12
eGFR	−3,59	−0,4	−3,14	0,001	−5,9	−1,29
NLR	193,78	0,6	5,72	0,001	125,56	262

eGFR: Estimated glomerular filtration rate; NLR: neutrophil/lymphocyte ratio.

**Figure 1:**
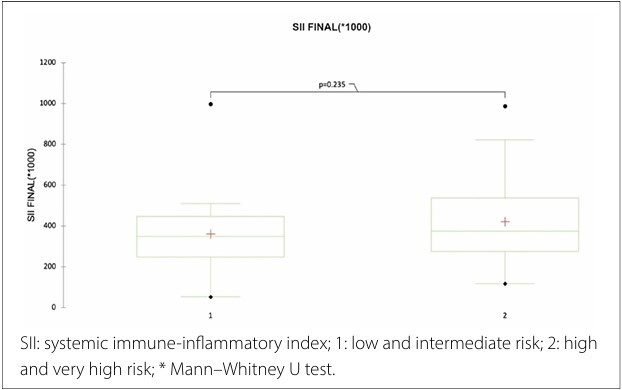
Relationship between the SII and Framingham risk score in patients with hypertension.

 As shown in **Figure 1**, the two groups (low-risk + intermediate-risk and high-risk + very-high-risk) were correlated with the SII. However, there was no correlation between the SII result and the Framingham risk score. 

## DISCUSSION

 In this cross-sectional study, adult patients with hypertension in a hypertension care program at a primary healthcare facility in Brazil were evaluated for traditional cardiovascular risk factors and their inflammatory parameters. The SII, a simple ratio based on platelet, neutrophil, and lymphocyte counts, positively correlated with age, renal function, and NLR in adults with hypertension. 

 Inflammation plays an important role in vascular damage in arterial hypertension due to the increase in oxidative stress.^4^ To this matter, many inflammatory biomarkers have been evaluated as surrogates to prevent undesired outcomes such as acute myocardial infarction and stroke. CRP, an acute-phase inflammatory protein, is associated with hypertension development when elevated.^14^ The NLR, a simple ratio between the neutrophils and lymphocytes, also showed a good correlation when elevated to predict arterial hypertension development in a Chinese cohort.^4,15^ Apart from the prediction role in hypertension development, the SII correlated with the development of left ventricle hypertrophy in hypertensive patients, showing the importance of inflammatory biomarkers in the prediction of hypertension and the development of structural damage once hypertension is installed.^16^ In our sample, the NLR was positively associated with the SII in hypertensive patients, suggesting that when elevated, hypertension might be present in patients. 

 Traditional risk factors such as diabetes, obesity, dyslipidemia, aging, male sex, and smoking are usually related to poor CV outcomes in hypertensive patients.^2^ In our sample, higher age was associated with a higher SII in hypertensive patients, but there was no correlation with sex. On the contrary, a South African study evaluated the accuracy of the SII to predict the new onset of essential hypertension.^17^ Women with higher age had higher SII results and developed essential hypertension in a higher proportion than men.^17^ Our sample enrolled 56% women; the majority were over 50 years old and above ideal weight. Aging is also associated with increased in blood pressure. A cohort study conducted in the United States of America evaluated the onset of essential hypertension with aging and aldosterone sensitivity.^18^ It was found that with higher age, the blood pressure levels are higher in patients with black ethnicity and in those with higher aldosterone sensitivity during the development of essential hypertension.^18^ The RAASi is one of the main targets for controlling blood pressure. Continuous RAAS activation induces organ damage,^19^ inflammation, and therefore, higher levels of SII. Most hypertensive individuals received an ACEi or ARB treatment strategy; however, we could not evaluate the rate of aldosterone inhibition because of a lack of financial support. 

 The CVD Framingham score was evaluated in our sample, and as it was shown, most of the patients had a high-risk CVD Framingham score.^12^ In a recent elderly Chinese cohort, the SII was evaluated as a prediction tool for clinical outcomes in acute myocardial infarction patients who had undergone percutaneous coronary intervention. The SII showed an independent predictive value for in-hospital mortality, in-hospital major adverse cardiovascular and cerebrovascular events, and long-term mortality.^20^ In our sample, the prevalence of acute myocardial infarction and stroke Arterial hypertension is associated with CVD, and the SII might be an efficient tool to predict future events.^5^ The early detection and control of blood pressure values are still the main clinical tools to avoid unfavorable outcomes.^21,22^

 There were some limitations to the study design and procedure. First, it was a single-center evaluation of patients in a hypertension care program. These patients may have had more information about their disease than the general population. Moreover, most participants used only two types of antihypertensive drugs with good achievement of the blood pressure goals, which reflects a less severe stage of hypertension. Owing to restrictions of the Brazilian public health system, a 24-hour arterial blood pressure monitoring device was not available to determine the range of blood pressure control. Adherence to drug treatment was evaluated, as was done in Brazil, using the self-report of the patient. However, using a single complete blood count to determine the ratio of neutrophils, lymphocytes, and platelets was revealed to be a good and inexpensive strategy to add information to the inflammatory status of patients. Usually, in the Brazilian Unified Public Health System, the requisition of hs-CRP and IL6 as screening tools is not permitted because of their high cost. This simple screening strategy could be adopted in low-income countries. 

## CONCLUSION

 In summary, in this cross-sectional study, the SII was positively correlated with age and renal function in patients with hypertension. Traditional risk factors for CVD include the addition of SII and NLR to address inflammatory status. 
